# Sword, Shield and Buoys: A History of the NATO Sub-Committee on Oceanographic Research, 1959–1973^1^

**DOI:** 10.1111/j.1600-0498.2012.00258.x

**Published:** 2012-04-05

**Authors:** Simone Turchetti

**Affiliations:** * Centre for the History of Science, Technology and Medicine (CHSTM), Faculty of Life Sciences, University of ManchesterManchester, M13 9PL, UK

**Keywords:** Cold War, forecasting, NATO, nuclear submarines, oceanography, Soviet Union, surveillance

## Abstract

In the late 1950s the North-Atlantic Treaty Organization (NATO) made a major effort to fund collaborative research between its member states. One of the first initiatives following the establishment of the alliance's Science Committee was the creation of a sub-group devoted to marine science: the Sub-committee on Oceanographic Research.This paper explores the history of this organization, charts its trajectory over the 13 years of its existence, and considers its activities in light of NATO's naval defence strategies. In particular it shows how the alliance's naval commands played a key role in the sub-committee's creation due to the importance of oceanographic research in the tracking of enemy submarines. The essay also scrutinizes the reasons behind the committee's dissolution, with a special focus on the changing landscape of scientific collaboration at NATO. The committee's fall maps onto a more profound shift in the alliance's research agenda, including the re-organization of defence research and the rise of environmentalism.

The oceans are vast. The United States Navy cannot do all the work by itself. We need the help of all of our friends and allies to help solve the riddle of 1 the oceans.^2^

During the Cold War the US investment in marine science surpassed that of any other country. But even this high level of research funding failed to reassure US administrators about what US oceanographers could accomplish without the support of international collaboration. The words proffered by the US Navy representative at the NATO forum for the alliance's naval commanders (see epigraph above) tell us much about this apprehension and the wish to obtain the assistance of the USA's closest allies in the pursuit of studying the sea. The administration's anxiety originated from the operations of nuclear weapons-carrying Soviet submarines and the realization that oceans, as operating environment, offered to these enemy vessels ‘concealment that couldn't be obtained in any other medium.’^3^ Charting this environment was no longer of importance only to the advancement of science; the definition of naval strategies and defence measures depended on it.

It is thus no surprise that the US-led defence alliance NATO was active in the support of oceanography; a commitment that led, amongst other initiatives, to the setting up of a Sub-committee on Oceanographic Research (ORC). Established in 1959 by the NATO Science Committee (SC), the sub-committee's formation was rooted in post-Sputnik preoccupations about catching up with the Soviet Union in the promotion of science and technology. Consistent with the SC mission, the subcommittee encouraged fundamental research through the offer of research grants to prominent scholars. Yet its distinctive trait was that the basic knowedge produced should be made pliable to the needs of NATO naval commands, especially that of improving the detection of submarines.

That NATO fundamental oceanographic research had military implications has already been suggested in the existing historical literature. John Krige, for instance, has argued that the sub-committee was born in an effort to promote research that ‘straddled the military/civilian divide’ (Krige, 2006, p. 206). This essay, however, provides a more in depth account of how the need to enhance NATO's monitoring of enemy submarines informed the ORC's history. As environmental conditions (sea temperature, currents and salinity) affected the performance of surveillance devices like sonars; NATO mobilized the Western marine scientists to prioritize the charting of these environmental factors in sea areas suitable for submarine detection. It was thus a specific defence urgency that propelled the sub-committee's activities.

By associating the scientific production of basic marine science and monitoring operations, this study contributes to the growing literature emphasizing how intelligence and surveillance needs shaped Cold War science. Since Paul Forman's pioneering study on the sponsorship of quantum electronics in the USA, we have learnt that the military exercised enormous influence over both the direction of research and whether researchers could retain their intellectual agenda in national sponsorship schemes (for alternative viewpoints see Forman, 1985 and Kevles, 1990). We have not, however, sufficiently investigated the motives behind this patronage despite the growth of contributions emphasizing these links. Fundamental research was decisive in securing new methods for the monitoring of (and intelligence gathering on) enemy forces (for an overview: Doel, 1997). The study of seismic waves allowed the detection of Soviet nuclear tests (Barth, 2003). That of the ionosphere was critical (and at times offered cover) to telecommunications; including the interception and decoding of enemy signals (Van Keuren, 2001). Military funding invigorated oceanography too, especially in the USA, due to its role in naval operations and the tracking of enemy submarines (Mukerji, 1989, p. 43 and pp. 73–74; Oreskes, 2003).

This study fills an important gap in our knowledge of Cold War oceanography by focussing on its promotion in a transnational research framework like NATO. It suggests that important episodes of international scientific collaboration were in response to surveillance needs as much as national research programmes. It also offers an opportunity to re-think NATO's patronage strategies. Krige has extensively analysed its implications for US/Western Europe relations and yet, according to Ronald Doel, scientific intelligence ‘lurks at the margins’ of Krige's account (Doel, 2010, p. 311). The archival evidence disclosed herewith suggests that the defence intelligence agenda was not at all peripheral to NATO sponsorship strategies, even if it was made less apparent by the alliance's science administrators in their bid to prevent criticism from national representatives less eager to endorse it.

The essay also aims to fill a gap in the historical study of 20th century oceanography. This literature has expanded considerably in recent years (Rozwadowski and Van Keuren, 2004; Laughton *et al.*, 2010), and there are studies that analyse cases of military patronage and international co-operation (Weir, 2001; Rozwadowski, 2002; Mills, 2009). But to date few of the authors have discussed the NATO sub-committee in sufficient detail. Jacob Hamblin is a notable exception. He claims that sub-committee's creation epitomized the disillusion of Western oceanographers with previous attempts at scientific collaboration (especially in the context of the International Geophysical Year, IGY, 1957–1958), and provided a collaborative context congenial to existing political alliances (Hamblin, 2005, p. xxi and pp. 231–236). Hamblin is correct in highlighting the tensions within the scientific community, but this paper shows that NATO's naval authorities exercised an equally significant influence.

The article is divided into three parts showing how the relationship between the production of oceanographic knowledge and the pursuit of surveillance of Soviet submarines evolved over time.^4^ It starts by discussing how the SC administrators decided to prioritize oceanography and establish a sub-committee devoted to its development in light of secret NATO information on future Anti-Submarine Warfare (ASW from now on) strategies. Then it highlights how, from 1960 to 1965, the sub-committee's research activities accommodated the surveillance needs that these strategies entailed. The paper's final section focuses on how changes in NATO defence and defence research removed these urgencies. In particular, the re-organization of naval coordination within the alliance that took place from the mid-1960s resulted in an increase in the funding of military oceanography, thus undoing the ties between the sub-committee and its naval sponsors. This new funding regime forced the sub-committee to look for a new research focus. Meanwhile, the alliance's science administrators instigated new studies on environmental and global problems (following the rise of Richard Nixon's ‘environmental diplomacy’). As the sub-committee had now to operate in a more competitive funding environment, these transitions anticipated its dissolution. Thus while looking primarily at the shaping of Cold War oceanography in connection with surveillance requirements; this paper also charts the transition from these needs to environmental analysis—at least within the NATO framework.

## 1. A Cosmic Top Secret

Why did the NATO Science Committee place oceanography as a priority item in its sponsorship agenda? We know now that in 1958 a representative of NATO's naval commands informed its members about top secret strategies typifying the detection of enemy submarines. These revelations were relayed in order to stir the committee into action by promoting oceanographic work that could support the improvement of naval surveillance operations.

This initiative was taken in a period when NATO navies lacked coordination and did not routinely exchange oceanographic information; a circumstance that concerned its naval commanders. Following the signing of the North Atlantic Treaty, on 4 April 1949, NATO's member states agreed on the definition of a joint naval command structure through the creation of the Supreme Allied Commanders for Europe (SACEUR) and Atlantic (SACLANT). A newly-established NATO Naval Steering Group ensured co-ordination between these organizations and national commands. These arrangements, however, did not immediately remove differences between the allies. Some of the NATO's navies had fought against each other during WWII. This mapped onto a broader disagreement between US and British administrations on naval coordination as the British government did not want the SACLANT to be a US Navy officer (Maloney, 1995, pp. 86–137). Other allies maintained dissimilar and problematic views. France developed an independent nuclear deterrent. The Icelandic parliament disputed whether the alliance should have a naval base on the island. Norway and Denmark did not allow nuclear weapons on their soil.

These divisions ruled out all-encompassing forms of naval coordination, including the sharing of oceanographic data of military importance, which only occurred between trusted allies. In 1957 the French Navy and the British Admiralty established an Anglo-French Naval Mutual Collaboration Project.^5^ In 1960 the US Navy agreed to share security-classified forecasting data for ASW purposes with Canadian and UK navies. From 1962 the USA, Britain and Norway collaborated on underwater research (Wright, 1999, p. 155).

But the information that Western intelligence agencies collected about the growth of Soviet oceanography suggested that these ad hoc alliances were inadequate. The lack of multilateral co-ordination was problematic. In 1952 US Navy reports revealed that the Soviets were about to develop a submarine-based nuclear deterrent. Their participation in the IGY, 1957–1958 confirmed that the Soviets had launched a major effort to chart the oceans, which heightened the Westerners' anxiety about the implications of this work, especially for submarine warfare. In 1959, the Directorate of Scientific Intelligence (British Ministry of Defence) published the confidential *Oceanography and Defence in the USSR, 1956–1958* revealing that Soviet oceanographers had initiated surveys in the Atlantic corridor and looked into its characteristics through systems of radio-controlled buoys. Although the intelligence officers downplayed the significance of what the Soviets had published (‘little more than a repetition of what has been done in the West’), they also suggested that critical data were withheld from publication for operational reasons.^6^ The SACLANT thus took responsibility for finding ways to co-ordinate oceanographic research within the alliance in light of these security concerns and notwithstanding the resistance of some NATO allies to data sharing.

By the time the British intelligence report was published, the North Atlantic Council had already approved the establishment of a Science Committee. The 1956 *Three Wise Men* document that informed its creation had outlined how the advancement of science in allied countries could function as a means to enhance economic, political and social integration.^7^ A 1957 US report titled ‘Trained Manpower for Freedom’ had also highlighted that Western Europe lacked programmes for the training of scientific personnel. The SC thus aimed to promote cultural integration and scientific training through a sponsorship programme that provided fellowships to individual researchers and support grants to research institutions. Its chairman acted as Science Adviser to the NATO Secretary General and its members represented either allied governments or military coordinating bodies, including the SACLANT (Krige, 2006, pp. 202–203).

While the SACLANT delegates viewed the SC mission in light of their current need to improve oceanographic co-ordination, the members of the new committee were divided the funding of research tied into defence problems. True as it is that defence research featured in the SC terms of reference, the committee experienced difficulties in taking forward a specific plan of action due to the objection of national representatives (Solly Zuckerman of Britain especially) worried about multilateral sharing of restricted data. Moreover, NATO science administrators had yet to make a decision about whether SC members could be granted the necessary security clearances. And finally a NATO group, the Defence Research Directors (DRD), already coordinated actions in this area. That said some national representatives in the committee, especially André Louis Danjon of France and Isidor I. Rabi of the USA, wanted to attune non-classified research to defence problems. In February 1958 a French proposal highlighted specific research areas, including oceanography, in need of support (Krige, 2000, pp. 98–99).

The following spring the debate on the SC research priorities came alive and US military authorities stated more openly their positions through the Standing Group (a sub-committee of the NATO Military Committee) and SACLANT delegates. They now advocated the promotion of fundamental research of use to defence problems, thus echoing the French and US delegates' viewpoint. During the first SC meeting of March 1958 the Standing Group representative, US Army General Theodore Parker, argued that notwithstanding the *Three Wise Men*'s recommendations SC activities ‘should be additional to, and not at the expense of, the military effort.’ The basis of the Alliance was ‘the need for collective defence’ and the military was ‘the leading customers for the end product of scientific R&D.’^8^ Parker thus wished that military problems found some space in the planning of NATO basic science.

During the second SC meeting (July 1958), the SACLANT representative produced a statement exemplifying Parker's viewpoint. US Navy Captain Kenneth M. Gentry (Deputy R&D Director for the US Chief of Naval Operations) informed SC members about NATO's strategy for tracking submarines thus revealing that oceanography was decisive to the future of NATO naval operations. As Gentry's account was summarized in a *cosmic top secret* document (i.e. secrecy ruling extending to all member states), it appears that difficulties with security clearances could be overcome; especially in light of a ‘vital and important’ subject for which the SC members' ‘full consideration’ was requested.^9^

**Fig. 1:**
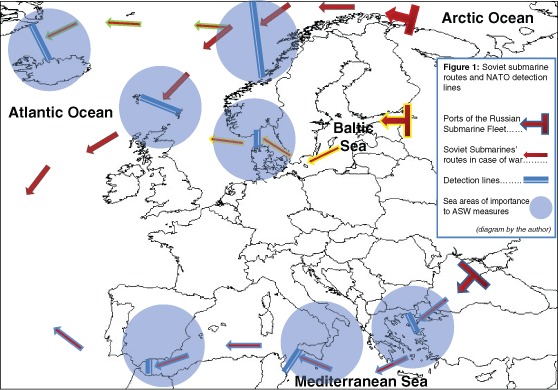
Ports of the Russian submarine fleet…
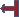
…and ‘choke-points’, i.e. points of access to Atlantic (and Mediterranean) waters 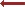.

In case of war, Gentry explained, the bulk of the Soviet submarine fleet in the Arctic, Baltic and Black Seas would head towards the Atlantic. NATO naval forces should block the submarines before they reached the ocean in order to limit their range of operation. SACLANT would use a tactic known as ‘destruction at source’ (eliminate the threat in their ports of exit: Leningrad, Murmansk and Sevastopol - with nuclear weapons if necessary), but these vessels were unlikely to be found at their bases. Western navies ought, therefore, to improve their ‘detection and kill capability’ in sea areas providing access to Atlantic waters.^10^ Gentry revealed that SACLANT aimed to improve surveillance of enemy submarines in key passages such as choke-points and narrow waterways (on this strategy see also: Harriett Critchley, 1984, p. 836). The allied commander wished to develop a system of monitoring vessels and stations along detection lines cutting across seven passages leading to the Atlantic: the transit between Greenland and Iceland, the Norwegian coast and the Faroe-Shetland Channel (from the Arctic), the Skagerrak strait (from the Baltic), Gibraltar, the strait of Sicily and the Turkish straits (from the Black Sea). Gentry concluded on the need for a surveillance system that turned ‘detection into a kill’ (see Figure 1).^11^ Monitoring, however, depended on a better understanding of environmental factors such as sea currents, temperature layering and salinity. Thus, implementing the new SACLANT strategy required collecting environmental data and knowledge.

Alerting a group of scientific experts dealing primarily with the promotion of science and technology to these strategic issues betrayed Gentry's wish to direct their action towards scientific problems of importance to new ASW measures. While the SC continued to debate, oceanography became a priority item for the NATO Science Adviser. After the second SC meeting Norman Ramsey drafted plans for a hazily defined ‘NATO oceanographic expedition’ and arranged a meeting of ad hoc experts for this purpose. The group met in Paris in February 1959.^12^

## 2. Only a Few Aboard

Ramsay carefully chose the invitees for the Paris meeting. He rushed to find oceanographers who could conceive a plan of action consistent with Gentry's statement and the experts either had important sponsors in the Office of Naval Research (ONR), or ties with oceanographers that its administrators held in esteem. The ONR, the largest naval research agency in the USA, provided technical advice to Gentry's organization (the Chief of Naval Operations) and routinely funded both restricted research and basic science in university laboratories. By then the organization was about to launch the largest national oceanography programme to date (Ten Years in Oceanography - TENOC) following the US administration's decision to prioritize a nuclear strategy based on the submarine-launched nuclear missile POLARIS. By inviting ad hoc experts to join the planning process, Ramsey offered no ammunition to those SC representatives that might have resisted prioritizing oceanographic work with ties to naval surveillance operations. Yet the experts' connections with the ONR suggest that Ramsey wished as much as Gentry to prioritize exactly these kinds of activities.

In the second half of 1958 the NATO Science Adviser alerted the Danish marine zoologist Anton Bruun about the forthcoming ad hoc meeting of experts. A leader of the post-IGY Scientific Committee on Oceanographic Research (that included marine scientists from Eastern and Western blocs), the Dane was better placed than Ramsey to consider the best experts for such a prominent cohort. But rather than letting Bruun take decisions about potential invitees, Ramsey gave him a list of oceanographers to be contacted.

At the top of that list was Columbus Iselin, director of the Woods Hole Oceanographic Institute (WHOI, Massachusetts, US). Iselin had pioneered the study of the Gulf Stream thus prompting research on oceanic circulation in the Atlantic. He was also influential in ONR sponsorship schemes to the point that the whole system ‘at times seemed almost incestuous’ due to the conspicuous funding of WHOI's programmes through ONR grants (Weir, 2001, p. 273). Ramsey openly stated the importance of having ‘on board’ an oceanographer endorsed by the ONR. If Iselin was unavailable, he wrote to Bruun, the American delegate ought to be a ‘US oceanographer sent by Admiral Rawson Bennett’ (the ONR Chief of Naval Research).^13^

The list included Iselin's closest collaborator in Europe: the British George Raven Deacon, director of the UK's National Institute of Oceanography (NIO, Wormley). Despite the contrasts between US and British naval commands, in 1957 Deacon and Iselin's institutes had initiated joint surveys with their research vessels *Chain* and *Discovery*. Not only had Iselin and Deacon pioneered the study of oceanic currents, but they had both been active in WWII anti-submarine work when the latter headed British Admiralty's Group W (Waves). Iselin investigated the effects of currents on sound transmission; also examining how submarines could find cover in currents by exploiting their different temperatures and become undetectable to sonar (Charnock, 1985; Hamblin, 2002, pp. 4–5; Mills, 2009, p. 225; Crease, 2010, pp. 118–119).

Iselin and Deacon's career trajectories show a much deeper synergy. They represented a tradition in marine science often referred to as ‘dynamical oceanography.’ Stemming from Vilhelm Bjerknes' work at the Geophysical Institute of Bergen (Norway), it informed oceanographic and meteorological studies forging a new generation of scholars (Friedman, 1993). Bjerknes' associates, Carl-Gustav Rossby and Harald Sverdrup especially, exercised influence in the American scientific community also contributing to ONR-funded underwater research (Mukerji, 1989, pp. 43–44). The third expert invited to the meeting, the Norwegian Håkon Mosby, was a prominent member of the same school. A veteran of oceanographic expeditions, the Bergen-based Mosby had, like Deacon and the other invitees, specialized in the study of currents and sea temperature layering (Mills, 2005, pp. 246–264; Roberts, 2010, p. 175 and 265).

The ad hoc experts had thus a history of collaboration, an understanding of their field's trajectories and important ties with the US naval research establishment. The list prepared by Ramsey also included Henri Lacombe of the Paris-based Laboratory of Oceanography. The ONR liaison officer in London, Robert Dietz, had indicated him as the ‘rising star’ in French marine science (Hamblin, 2005, p. 65).

During the meeting of 25–26 February 1959 at NATO headquarters in Paris, the invited experts proffered what the SC delegates wanted to hear. Aware of the differences and variety of interests represented within the alliance, they did not exclusively emphasize the military implications of oceanographic research. They stressed instead that co-operation between European nations increased the effectiveness of Western oceanography. They paid lip service to the SC members by envisaging a variety of ‘important problems,’ and not just ASW, to which oceanography offered solutions. These included: ‘fishing, meteorology, submarine operation, ocean transportation, effects of radioactive fallout and waste, anti-submarine warfare.’ Yet we have seen that only the last one was introduced in a top secret SACLANT briefing, while none of the others were discussed in the same detail. The oceanographers' conclusions recommended the establishment of a research committee, rather than just the setting up of an expedition, effectively calling for some degree of autonomy in funding management and decision-making.^14^

These experts, however, openly stated the defence implications of oceanography in the presence of NATO naval commanders. In June 1959 the SACLANT established an ASW research centre, SACLANTCEN (now Undersea Research Centre), at the naval base of La Spezia, Italy. Directed by American nuclear physicist Eugene Booth, the centre became a focus of research on propagation of sound underwater and innovative acoustic detection methods (Wright, 1999, p. 155. See also Allan, 2008; Ranelli, 2008). A few months before the first meeting of the SACLANTCEN advisory council, Iselin urged Deacon to attend it. On that occasion the British expert highlighted the implications of oceanography for sonar detection and tracking of enemy vessels.^15^ After the meeting Deacon received a draft report from the NATO deputy science adviser in which the defence aspects of oceanography were openly laid out. Oceanographers ought to understand the physics and layering of oceans in the same way in which radar and radio physicists had looked into that of the ionosphere to improve accuracy in telecommunications and tracking of enemy aircrafts.^16^

In July 1959 the North Atlantic Council approved the ad hoc experts' recommendations. The SC now agreed that a sub-committee should be established, and indicated that the ad hoc experts be nominated ORC members. Meanwhile, the Defence Research Directors (DRD) endorsed Ramsey's proposal that oceanography should be singled out as a research area with implications for defence problems and suitable for international co-operation.^17^ The decision effectively sanctioned that some fundamental research (also including operational research, meteorology and defence psychology) would be funded because it allowed a concerted effort on research items of military significance. The DRD recommendations also made it possible for the SACLANT to request the ORC members' scientific advice when needed.

These decisions allowed Rabi to be a little more open in the SC context about the implications of oceanographic work. In a statement at the SC meeting of September 1960 he argued that the ORC programme should be given ‘very high priority’ and advocated ‘the closest liaison’ between the ORC and SACLANTCEN at least in an effort to avoid duplicating work.^18^ By then the NATO Science Adviser had succeeded in casting the ORC programme within the SC mission by calling in ad hoc experts during the planning process and letting them explain the variety of benefits to be derived from launching a NATO oceanographic programme. Yet the analysis of these experts' background and previous collaborations indicates that priority in this programme would be given to ASW-related problems and that such a research focus would also instigate collaboration with SACLANTCEN beyond the task of making sure that the same work was not replicated. Not surprisingly, it was exactly these kinds of activities that were given precedence, as we shall now see.

## 3. Surveys, Buoys and Hindcasting

In the 5 years that followed its establishment, the ORC recommended the funding of projects focussing on three endeavours: the accomplishment of surveys; the design and production of novel oceanographic equipment; the completion of investigations on hindcasting (the forecasting of sea phenomena through computational methods). These projects marked the expansion of physical oceanography in Europe and ensured furtherance of research previously carried out by the ORC members in other national or collaborative frameworks. The comparative analysis of this research programme and Gentry's document also reveals that NATO oceanographers re-directed their research interests to accommodate the surveillance concerns expounded in the top-secret statement. In particular, the surveys focussed on the sea areas surrounding the detection lines discussed in Gentry's document; novel monitoring equipment installed on buoys assisted both surveying and surveillance operations in some of these key areas, and hindcasting research indicated those sea areas suitable for enemy submarines' concealment tactics.

By 1965, when the ORC completed its first programme summary report, the sub-committee had designed 22 projects, employed 65 scientists in the establishments of participating institutions and published 19 technical reports. Between 1960 and 1964 it received funding of $700k (US dollars) and a further $300k from national organizations.^19^ While funding of specific projects ought to be officially ratified by a NATO research grants advisory panel, the ORC projects were earmarked for support before the panel met.^20^ In actual fact this sponsorship mechanism allowed the sub-committee's members to suggest innovative projects and receive grants to co-ordinate them. In 1960 US physicist William Nierenberg, who had previously worked on underwater detection problems, became NATO Science Adviser (Oreskes, Conway and Shindell, 2008) thus increasing even further NATO support for physical oceanography. Several new members joined the sub-committee, including the marine zoologist André Capart (Belgium's *Institute Royal des Sciences Naturelles*) and the oceanographer Walter Hansen of the University of Hamburg (Germany).^21^

**Fig. 2:**
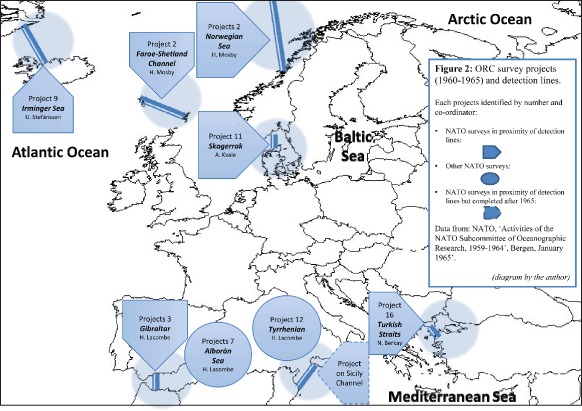
ORC project for oceanographic surveys. Source: NATO ORC, *Activities of the NATO Subcommittee on Oceanographic Research, 1959–1964*, Technical Report 20, Bergen, 1965.

Oceanographic surveys represented the largest expenditure and provided environmental analysis on detection and kill passages. NATO oceanographers collected data on currents, temperature layering and water exchanges between seas adjacent to these straits (Figure 2).^22^ They used the vessels of participating institutions: *Chain*, *Discovery*, Bergen's *Helland-Hansen*, the French *Calypso* (famously utilized by Jacques Cousteau). SACLANTCEN collaborated with the ORC on a number of surveys with its research vessel *Aragonese* (Ross, 1980, pp. 19–22).

**Fig. 3:**
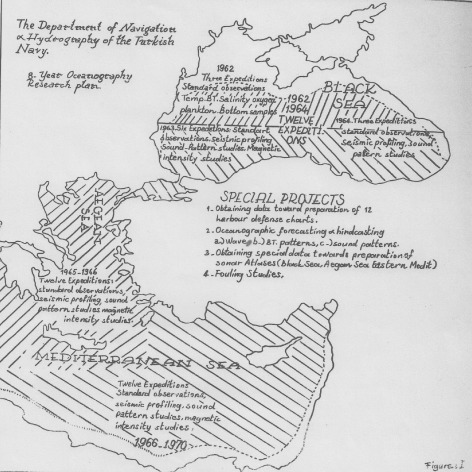
Plan for ‘special projects’ designed by the Turkish Navy's Department of Navigation and Hydrography in collaboration with the US Navy. Source: Essentials of the Project Supported by the NATO Research Grant. Turkey, 12 July 1962, Box 87, Deacon Archive (Reproduced courtesy of the National Oceanographic Library, National Oceanography Centre, Southampton, UK).

Mosby, who chaired the sub-committee up until 1966, had looked into the dynamics of Norwegian Sea currents for several decades and NATO funding helped him to further these studies with two projects on this sea and the Faroe-Shetland Channel (Mosby, 1963).^23^ Lacombe coordinated the Gibraltar strait project and similar surveys in the Alboràn and Tyrrhenian Seas. These two projects stretched surveying activities beyond sea areas envisaged in Gentry's document, but they also complemented SACLANTCEN work on sound propagation (Ross, 1980, pp. 12–19). All the other surveys carried out in this period hit on the detection lines envisaged by Gentry. And when an ORC member suggested the exploration of sea areas of less strategic importance, the proposal was not implemented. For instance, the Icelandic oceanographer Unnsteinn Stefánsson wished to explore the passage between Scotland and Iceland because of its significance to fishery studies. But his proposal strangely disappeared from the minutes (something he complained about with Deacon).^234^ Stefánsson eventually became co-ordinator for the Irminger Sea survey (the strategically vital Iceland/Greenland passage). In the case of the Turkish straits survey, not only did it hit on one of these critical ‘choke-points,’ but it complemented special gravity and magnetic surveys jointly carried out by the ONR and the Turkish Navy's Hydrography department. Turkish oceanographers trained in the USA used the same vessel for the ORC project and this collaborative framework (Figure 3).^25^ Gentry's document mentioned only one passage that the sub-committee did not explore before 1965: the channel of Sicily. A survey of this passage was completed 2 years later.

Furtherance of basic oceanographic science assisted ASW measures also through the design of state-of-the-art oceanographic instrumentation and the provision of systems of measurements and automatic recording of environmental data. One ORC project aimed at laying out a system of moored buoys in the Faroe-Shetland Channel. Odd Dahl (*Christian Michelsen Institutt*, Bergen) co-ordinated the project together with Mosby. Dahl recruited the Norwegian Ivar Anderaa to design an instrument for measurement and data collection that was completed in 1964 and trialled during ORC surveys. The instrument had sensors to calculate speed and direction of currents, temperature and salinity. A fixed buoy structure tied to the seabed assembled several of these devices at different depths (Dahl, 1969). The buoy project was funded at a cost of $50 k per year.^26^ It also stimulated interaction with the private sector as, thanks to the collaboration between Bergen's and Wormley's institutes, the ORC secured a deal with the British firm Plessey for the production of a commercial device: the Anderaa RCM4 (Crease, 2010, p. 125; Gould, 2010, p. 131).

The moored structures containing these sensing devices had value as scientific installations, but they also addressed the urgency stressed in Gentry's document to ensure the collection of intelligence on the transit of ships (and submarines) across passages. Buoy installations offered ideal cover for surveillance operations. In 1959 British intelligence had exposed the existence of a Soviet buoy array also utilizing automatic recording of oceanographic data. From 1961 the US Navy had made operational its Sound Surveillance System (SOSUS), an array of hydrophones covering the underwater areas surrounding the USA. Such a system, however, ensured no coverage beyond the Eastern Atlantic, thus compelling the US and its European allies to provide coverage for the sea areas closer to the continent. In the same year the British Admiralty representative at the NATO Naval Steering Group, informed colleagues that his organisation was busy designing a new type of buoy for the identification of submarines also calling for international co-operation in this research.^27^ This is exactly when the ORC began trial installations with the Anderaa device in the Faroe-Shetland channel; the main exit passage for the Soviet fleet in case of conflict. The function of these buoys was the collection of environmental data, but they were equipped with additional sensing devices such as radar and sonar and radio-equipment for the transmission of recorded environmental and ‘non-environmental’ information. The Soviet Union responded to these NATO efforts by installing a similar buoy system of surveillance along the sea passages stretching from Greenland to the UK (the so-called Greenland-Iceland-UK/GIUK Gap). These installations became the subject of CIA studies as its purpose was precisely to monitor the movement of nuclear submarines.^28^ Soviet trawlers were also busy sabotaging or stealing Western allies' buoys, something that instigated collaboration between the NIO and the British Ministry of Defence (Hamblin, 2005, pp. 187–188). Thus, while gathering data on environmental factors, NATO buoy installations offered important information on the movement of ships in general and underwater vessels more specifically.

Surveys and buoys, however, made the ORC no more than an instigator of prosaic data collection, whereas its ultimate goal consisted of using the records in the design of environmental prediction methods. Gentry's document had revealed that Soviet submarines could find cover in North Sea fjords and tidal movements made these coves suitable or unsuitable locations for the hiding vessel. Thus the ORC promoted innovative research focussing on swells and storm surges and, unsurprisingly, the North Sea was targeted as the key sea area for hindcasting studies.

Since WWII the military advantages to be derived from a more accurate prediction of weather events had become apparent. British and Norwegian meteorologists provided accurate prediction of meteorological conditions and tidal movements on occasion of the allied troops' landing in Normandy (Fleming, 2004). From 1946 US-Hungarian mathematician John Von Neumann pioneered, through the ‘Meteorology Project,’ the application of numerical and computational methods to weather prediction using the mainframe computer ENIAC (Harper, 2008, pp. 104–198). Hindcasting research complemented these studies and offered similar returns in knowledge to organizations responsible for planning naval operations. The ORC hindcasting project, directed by Hansen, consisted of designing numerical methods to predict North Sea surges. From 1964 statisticians Hans Erik Jensen and Steen Weywadt of the Institute of Mathematical Statistics and Operational Research at the Technical University of Denmark designed a computer model for their forecast (Jensen and Weywadt, 1966). By then two European institutions, the Tidal Institute (University of Liverpool) and *Institut für Meerskunde* (University of Hamburg) had pioneered numerical prediction methods through the study of residuals (difference between predicted and observed sea levels) and hydrodynamical equations applied to set parameters (Carlsson-Hyslop, 2010, pp. 287–300). While there is no reason to suggest that this type of research was tied exclusively to naval defence goals, it is worth considering that the US Navy ASW prediction system only covered the Atlantic and Arctic oceans. There was nothing offering a similar level of information and accuracy for the North Sea. Moreover, during one of the NATO Naval Steering Group meetings and in the presence of naval officers, Lacombe had provided evidence of the importance of supporting hindcasting work in light of its decisiveness in submarine detection.^29^

As the ORC programme was re-directed so as to provide data and analyses badly needed by NATO's naval commands, ORC members neglected the funding of projects which had less relevance to naval operations. Unsurprisingly marine biology was under-represented in the ORC framework: one project co-ordinated by the Italian marine zoologist Umberto D’Ancona (Hydrobiological Station, University of Padua), focussed on fish productivity in the Mediterranean.^30^ Moreover, the sub-committee never paid sufficient attention to the provision of fellowships for training, in contrast with what represented the SC's key mission. ORC project number one (Research Associates) aimed to offer scholarships to young trainees, but the oceanographers used the allocation almost exclusively to employ qualified personnel for the ORC surveys.

This is because the oceanographic sub-committee paid attention to the production of fundamental knowledge useful to NATO naval commands rather than the training of scientific manpower. From 1960 the NATO oceanographers participated in the Naval Steering Group's meetings where they considered the strategic implications of their work together with naval officers, also planning future activities. Lacombe reiterated that closer scientific co-ordination in marine science had value for the allies' navies as it represented a concerted attack on oceanographic phenomena.^31^ His words echoed those of the US Navy representative, who argued that such an effort was decisive to the allied navies' mission due to the ‘circular pattern’ that typified the dissemination of oceanographic information:

…military operations require certain data which are acquired in surveys. These data are interpreted and analysed, then disseminated back to military operations, ships, instruments, facilities and personnel [which, sic] are required to analyse and disseminate the data. A feed in is also given to designing ships, equipment and weapons systems. Of course basic research feeds into everything by improving our basic knowledge.^32^

In 1961, the NATO committee on long-range planning chaired by Theodore Von Karman noted repeatedly that ‘improved understanding of oceanographic phenomena depends on the advance of fundamental research which must be supported by the military […] The development of oceanography must be accelerated if we are to meet future military requirements’ (cit. in Ross, 1980, p. 19). And 3 years later even the otherwise chary British Admiralty officials pledged to pay ‘considerable attention’ to ORC work that was used to develop synoptic oceanographic forecasting and to ascertain detection ranges.^33^ So, at least up until 1965 the production of basic oceanographic knowledge through the ORC activities served a specific function within the NATO naval command structure as this knowledge was distributed amongst specialists in naval organizations and used at their end for operational purposes.

But by the mid-1960s this framework of activities changed considerably, as NATO defence research underwent an important reorganization. While ORC work on the key sea areas envisaged in Gentry's document reached completion, this restructuring placed the production of fundamental oceanographic knowledge that tied into surveillance requirements under a separate sponsorship system now encompassing solely defence research groups. Lacombe was correct in advocating a concerted effort, but he did not know that the ORC would no longer be among the organizations called upon to carry it out.

## 4. Clouds on the Horizon

From 1965 NATO defence planners advocated a major restructuring of naval strategies so as to allow oceanographic information to be disseminated more regularly amongst NATO and national naval commands. These transitions strengthened the ties between national defence research groups undoing those between SACLANT and the ORC. In turn the SC delegates reconsidered the independence and financial autonomy that was originally granted to the ORC due to the importance of its mission to the success of naval operations. The sub-committee's activities now attracted growing criticism.

Following the Cuban Missile Crisis, NATO military planners reviewed defence plans to accommodate a new strategy of ‘flexible response.’ The Superpowers' confrontation on the deployment of nuclear missiles in the Caribbean island had shown how problematic the strategy of massive retaliation was, thus envisaging the need of a more versatile and diversified response to avoid ‘mutually assured destruction.’ Flexible response, according to a NATO Military Committee report, ‘made it imperative’ that military oceanographic information was made available to NATO commands at the onset of an emergency because naval forces needed it to track down and identify enemy vessels before they launched the weapons they carried.^34^ Moreover, the creation of a SACLANT-controlled nuclear submarine fleet made it necessary to reconsider security criteria in the distribution of oceanographic data. The Naval Steering Group thus revised the alliance's dissemination policy and the SACLANT now took responsibility for ASW data of utility to NATO. The allied commander also set up data centres in Northwood (UK) and Malta and, from 1965, appointed a NATO military oceanographic agency (MILOC) to explore more closely the determination of sonar ranges in light of the sea current patterns that the ORC investigated. MILOC would also execute magnetic and gravity surveys.^35^ Given that by 1965 most ORC surveys were about to be completed and the new data dissemination structure offered a wealth of information to NATO naval forces, the need for the ORC to focus on research items of interest to naval commands was greatly reduced.

The restructuring process affected the interaction between NATO civilian and defence research too. In 1967 the DRD became the only permanent committee advising the alliance's military organizations.^36^ A DRD Exploratory Group had by then concluded that the NATO Science Adviser's action had not stimulated military-related science sufficiently, and thus the SC should no longer prioritize international cooperation in defence-related areas (Krige, 2006, pp. 206–207).^37^ It is unclear if the criticism extended to the oceanography sub-committee's activities given that the Naval Steering Group had actually praised its work. Yet responding to a Science Adviser's request for clarification on how military groups would seek advice from the alliance's scientists in the future, the SACLANT now indicated that it no longer needed ORC's assistance. The sub-committee may be consulted, but more sporadically than before.^38^

These transitions presented the SC members with an opportunity to re-consider the sub-committee's financial autonomy and research agenda. In June 1963 the Canadian representative urged his colleagues to re-examine the ORC position opposing the idea that ‘any single project […] should be continued indefinitely.’^39^ The following December he stressed that NATO defence organizations, rather than the SC, ought to support oceanography ‘projects of specific military significance.’^40^ The Canadian's criticism drew on an on-going polemic. Fostering oceanographic research under the aegis of a strongly defined political entity like NATO affected the efforts of non-NATO oceanographers to bring the Soviets into other international cooperative schemes. The British marine biologist Arthur J. Lee, of the International Council for the Exploration of the Sea, had voiced these concerned and clashed with Deacon on several occasions, also criticizing the ways in which the sub-committee disseminated his findings (Rozwadowski, 2002, pp. 206–207; Hamblin, 2005, pp. 232–235).^41^

From 1965 the polemic stirred by the Canadian representative extended to other items in the sub-committee programme, especially because NATO oceanographers appeared to be unrestrained in the use of funding. The ORC budget had by then risen well over its yearly allocation of $200k per year. The Research Associates project continued to grant no fellowships for training, but its cost rose from $25k (1964), to 30k (1965), to 47k (1966). This and other ORC projects were now frowned upon. In 1967 SC representatives from Norway and France argued that the hindcasting project taxed too heavily on the science budget.^42^ In the same year the chairman of the NATO research grant advisory panel complained that oceanography represented an ‘inertial mass’ taking 25–30% of the resources available for new fellowships. French and German representatives thus suggested reducing the sub-committee's financial independence by putting its budget under SC control.^43^ As old ORC projects reached completion, new ones could not be so easily approved because the SC now wished to use funds sparingly. In 1969 only 14 out of 28 grant requests were recommended for support. In 1970 the SC obliged the ORC ‘to work within a maximum ceiling to its total budget.’ Its secretariat was disbanded.^44^

Meanwhile the SC members debated if NATO should give precedence to military or civilian science. During the meeting of February 1965 Rabi clashed with the UK representative, Alan Cottrell, over the sub-committee's future as the latter asserted that the alliance should now prioritize collaboration between military research groups rather than synergies between civilian and defence science. British diplomats at the NATO headquarters commented that cuts in the SC budget represented ‘a good opportunity to do a little streamlining,’ and that only ‘Military Science’ should be supported.^45^ The altercation reflected changes in the SC's financial structure. Until then its funding had predominantly come from the US administration and in 1965 the introduction of the ‘burden-sharing’ formula (based on Gross National Products; see Krige, 2000, p. 96) increased the contribution due by America's allies. Thus, if until then Britain's representatives at NATO had complied with the US wish to fund fundamental research; they now uttered their opposition because further investments entailed a more generous UK contribution.

Some US-allies now claimed that SC needed streamlining and that the ORC represented a load on it, which meant that the sub-committee was now believed to be a financial burden ‘squared.’ The oceanographers needed a new plan of action if their organization was to retain a role in NATO despite its recent shake-up. But NATO oceanographers divided over the sub-committee's future. Deacon couldn't avoid considering his government's call for financial restraint and Iselin was by then struggling with ageing and alcoholism (Stommel, 1994, p. 180). Mosby thus took responsibility for a new and ambitious programme and in 1968 set up an ad hoc oceanography group with the hope of finding a new research focus for the sub-committee.

## 5. A Changing Environment

In the late 1960s the NATO science programme changed considerably in terms of focus and allocations. The alliance began to play a different role in global affairs with the rise of environmentalism and its policy-makers' attempt to build a dialogue between East and West. Meanwhile, the reduced investment in NATO science paved the way for a new policy privileging the allocation of smaller grants for several new research cohorts. While the ad hoc oceanography group managed to find a new research focus enabling the ORC to set an agenda consistent with this new science policy framework, the new NATO economic regime ruled out support for the large-scale endeavours the oceanographers had in mind. Increased competition for NATO sponsorship and financial constraints thus defined a new situation that made it more difficult for the ORC to retain a role within the alliance.

In 1969 the ad hoc oceanography group met seven times. In 1967 Capart had succeeded Mosby as ORC chairman. But the Norwegian oceanographer, as the co-ordinator of the ad hoc oceanography group, took responsibility, together with Dahl, Lacombe and WHOI's Allyn C. Vine (see Oreskes, 2003), to design the sub-committee's new research agenda.^46^ They concluded that the ORC should now focus on air-sea interaction as such analysis could be used to help explain global patterns of weather change. They thus advocated establishing a North Atlantic Platform; a large manned buoy/vessel structure that could collect data through a variety of sensors.^47^ By then, similar facilities such as the French Bouèe Laboratory and the ONR buoy/vessel FLIP (FLoating Instrument Platform) already operated in the Mediterranean and the Pacific. Notably, NATO oceanographers evidenced the platform's utility to both environmental analysis and surveillance activities thus tying together old and new ORC priorities. The platform could be used by climatologists and biologists (monitoring of plankton), as well as for traditional ASW buoy operations including ‘*veille surface*’ through radar and ‘*veille sous-marine*’ through sonar.^48^

The platform's cost would however be well above anything requested that far by the NATO oceanographers. When the Science Adviser received the proposal, he manifested some reservations. The Norwegian Gunnar Randers, formerly a science planner in the Norwegian atomic and defence research establishments, shared Mosby's view that the ORC should formulate a more focussed programme of activities.^49^ But he worried about the expenditure, especially as there was resistance among the European allies to burden-sharing. In March 1970 Deacon had written to him saying that it was ‘too soon to think of putting a lot of money into a large North Atlantic Facility.’^50^

Randers chaired the newly-established NATO Committee on the Challenges of Modern Society (CCMS); a circumstance that made him even more wary about offering support to the oceanographers' ambitious plan. Launched in April 1969 by US President Richard Nixon, the CCMS aimed to tackle environmental and global problems through innovative research. Of course Nixon's environmental diplomacy chimed with his domestic political goals, especially in light of the recent protests on university campuses and the Vietnam conflict (Hamblin, 2010; Turchetti, 2010). But it also marked a clear shift in NATO's patronage strategies. In particular, the CCMS budget originated from voluntary national contributions (in contrast with the SC's cost-sharing mechanism) thus ensuring the promotion of innovative research at no extra-costs for the alliance.

Unwilling to support large-scale endeavours in this new NATO financial framework, Randers appointed a special consultant on oceanography so that the platform project could be divided into segments. The consultant Amos J. Shaler, a metallurgical engineer who had advised SACLANT and MILOC, used network analysis to assess the sub-committee's plans. He viewed favourably the development of buoy-based systems for environmental studies and their application to a variety of problems including surveillance, marine pollution and seafood supplies. His report outlined the scientific problems that needed to be tackled and the resulting benefits on the micro-, meso- and macro-scales.^51^

NATO oceanographers, however, dismissed its utility. During the sub-committee meeting of 21 October 1970 the Canadian Neil J. Campbell noticed that some of the micro-scale studies were outside the ORC terms of reference. So were meso-scale studies regarding pollution problems; Lacombe argued. Deacon stated that the study did not shape a programme of actions.^52^ Shaler's review irritated the oceanographers mainly because it was forced upon them by Randers. Yet the Science Adviser had acted in the knowledge that unless the ORC succeeded in designing a plan of action in agreement with the new NATO sponsorship regime, its mission would be too indistinct to be supported by the alliance in the future.

It was now apparent that the sub-committee was at risk of being shut down. During the meeting of 5 April 1971 a minute of silence commemorating Iselin's death preceeded Randers' outcry. The Science Adviser stated that the ORC's value was ‘as apparent as ever,’ but its budget would suffer due to competition from other NATO organizations.^53^ He thus reiterated the need to follow Shaler's advice and plan its activities. If the oceanographers agreed to these intentions, he was even prepared to reinstate oceanography as a separate item in the science budget. When the oceanographers rejected the proposal, Randers envisaged the opportunity of a merger with the NATO meteorology group, which shared with the ORC an interest in air-sea interaction studies. But in 1972 he put forward a far more drastic plan: the sub-committee would cease its activities at the end of that year.^54^

Randers' draconian measures might have been taken in light of the disagreement with (and between) the oceanographers, but it is also likely that the administration of ORC grants, which had attracted criticism at the NATO headquarters, played a part. Notably, the groups that replaced the sub-committee enjoyed far less independence and financial support. An air-sea interaction panel, chaired by NIO's oceanographer Henry Charnock, had Lacombe amongst its members and aimed to complete work originally designed by the ad hoc oceanography group. Meanwhile the special programme panel on marine science, that included Deacon and Campbell amongst its members, set out to use small budgets to give ‘maximum catalytic effect’ to innovative research in marine biology.^55^

## 6. Conclusions

The ORC history shows that the circumstances of its patronage affected both its ascendancy and fall. The ASW requirements offered Western oceanographers a ‘context of motivation’ (Oreskes, 2003) to prompt fundamental research. The establishment of an organization devoted to marine science addressed the US anxiety deriving from the growth of Soviet oceanography, the surveillance requirements of the alliance's navies and the shortcomings of naval coordination between its member states. The US and its Western European allies were not prepared to share defence data. The prospect of a forum that would produce open and unrestricted knowledge represented a solution to the problem of gathering and sharing oceanographic information. The knowledge produced by the sub-committee could be applied to a variety of problems, but a naval urgency prompted the effort to put it together. In particular the emphasis on the study of choke-points in the Arctic, the Baltic and the Mediterranean derived from the need to support new detection measures for surveillance of Soviet submarines entering Atlantic waters. Thus NATO's ‘sword and shield’ strategy found implementation at sea through the use of far less iconographic floating devices serving both as tools of surveillance and scientific instruments.

The oceanographers who played a key role in the ORC used the circumstances outlined by the ASW requirements to significantly expand the field in Europe. NATO sponsorship also helped them to further studies on the dynamics of oceanic circulation that existed from much earlier. Moreover, the management of new research increased their reputation in the scientific community, even elevating some of them, like Deacon, to a leading role. In turn this allowed Deacon to challenge the Soviets internationally and clash with British colleagues wishing to instigate collaboration with them, thereby carrying out ‘oceanography without an apology’ (Hamblin, 2005, p. 172).

It is somewhat ironic, however, that while Deacon so forcefully pursued these goals, the power that helped him to achieve them evaporated. From 1963 the reorganization of defence research at NATO set the conditions for a review of the committee's activities. It is possible to speculate that the ORC work did not satisfy the SACLANT, in which case the naval commander's decision to renounce support for the sub-committee derived from this disappointment. Yet, SACLANT's pronouncement was not the only factor determining this revision. By the mid-1960s the SC no longer wanted to accord the financial and research autonomy that the sub-committee had enjoyed since its establishment. From 1969 the growing influence of environmental diplomacy set the conditions for increased competition in funding allocation. The following year the project for a North Atlantic Platform failed to materialize due to cost concerns. A review of the sub-committee's activities soon followed and then its dissolution.

While this account on the ORC's history resonates with other studies on the history of Cold War science, it sits uneasily with dominant historiographical interpretations, in particular with respect to the circumstances of patronage in a transnational framework like NATO; the implications of oceanographic research for surveillance operations; and the role of Cold War science in the shaping of environmental analysis.

It is questionable whether this case validates further Forman's ‘distortionist hypothesis.’ No oceanographer was openly co-opted to develop a specific plan. While the SC members knew about oceanography's role in the shaping of future ASW measures, no explicit request appears to have been made by the representatives of naval commands for specific actions. Moreover, the funding granted to the ORC might well have been ‘soft-money’ in terms of size of investment, but at least up until 1967 NATO oceanographers administered it independently, enjoying the freedom to carry out the research they wanted to pursue (on soft-money see: Mukerji, 1989, pp. 52–53). It is also true that an ‘anti-distortionist’ interpretation focussing on the ability of oceanographers to retain their intellectual agenda might equally fail to incorporate this case (see for instance Kevles, 1990). The sub-committee's circumstances after 1965 show that NATO accorded support to the oceanographers' programme for as long as an interaction between civilian and military (naval) research was sought. Its reduced weight in NATO affairs forced the oceanographers to operate in a less profligate funding environment.

On the whole the ‘mechanics’ of patronage appear in this case to have been somewhat more subtle. The experts were metaphorically given a white canvas on the understanding that their interests would lead them to paint in the way desirable to SACLANT—or at least to grant their independence for as long as they did so. What counted therefore was not their direct input in selecting research items to work on, but in picking up the right people for the tasks ahead; notably all oceanographers with a reputation at the ONR. Presumably informal (and non-documentable) ties between sponsors in NATO naval organizations and scientists might have been decisive in casting these plans.

If this is the case, then these experts appear to have been interpreters of a new research agenda rather than managers of a set plan. This evidences even more their ability to dwell in both the scientific and diplomatic arenas and to gain and retain control over their programme. This is even more relevant in a transnational space like NATO where a variety of vested interests existed and found representation through national delegations, military bodies and civilian agencies. The US administration, through its military (Parker, Gentry) and civilian (Rabi) representatives, continued to urge its allies to strengthen the co-ordination between civilian and military science. French and Norwegian administrations had similar interests. Britain (and partly Canada) had a more ambivalent approach revealing the wish to maximize returns in knowledge while attempting to reduce costs and threats deriving from data dissemination. The NATO oceanographers reflected these various positions. When the committee was first established, these experts were perceptive enough to emphasize the value of oceanography for problems beyond ASW so as to accommodate these different views. And free as they were, their action was ultimately tuned in to that of their governments. Mosby and Lacombe continued to pursue oceanography through the promotion of large-scale endeavours consistent with the sizable investment that their governments granted to marine science. By contrast, from the mid-1960s Deacon might have realized that the new funding environment did not allow him to carry out oceanographic studies in an ‘un-apologetic’ fashion. He thus merely reiterated instructions received from London; he was urged to make sure that NATO funding was used parsimoniously.

It is also important to underscore the role of NATO Science Advisers in finding a compromise between these different (and often diverging) interests; at times successfully (Ramsey) but sometimes unproductively (Randers). In particular, their actions aimed to blend different agendas given that the directions of the NATO science programme polarized national delegations. Finding a solution to these contrasts entailed official decisions whose implications, however, could not always be officially stated. Ramsey appointed *experts* to provide indications about the future oceanography programme, yet these were oceanographers skilled in ASW studies or endorsed by the ONR. Randers appointed *a consultant* to review the ONR programme, yet the appointment aimed to justify a cost-cutting exercise.

NATO's science policy was always driven by personality (and idiosyncrasies), but it encapsulated political, strategic and military urgencies. It was consistent with the USA's quest for hegemony in Europe as evidenced by the sharing of cultural ideals and the exportation of a American model of training and research (Krige, 2006). But it also created an opportunity to address important tasks of military coordination and transfer a US national security agenda within the dimension of international scientific collaboration. During the Cold War, environmental knowledge became a key resource for intelligence and surveillance operations. US administrators, however, felt their country was not up to the challenge of gathering of environmental data alone. Following the IGY, the SC establishment offered an invaluable opportunity to put international collaboration to work in a new context.

Oceanography occupied a special place in the USA's attempt to inform collaborative work with a national security agenda due to the oceans' vastness and intimate complexity. Fundamental research delivered a wealth of new data and projects focussing on basic science did not compromise classified research. It may well be that this open knowledge assisted allies and enemies alike, but its real value rested with the possibility of integrating the new data in further synoptic work separately carried out by naval defence research groups. It is also likely that alternative channels of dissemination for the results of this work existed as ORC hydrographic data were made available to World Data Centres, but technical reports were issued in limited numbers and made available on request; a dissemination practice that attracted criticism in the community of oceanographers. In any case, the NATO marine scientists do not appear to have constituted a ‘reserve labor force’ as their programme was instigated by the need to strengthen international cooperation and not the training of scientific manpower. Their activities fell in a ‘grey area’ between civilian and military research; like other ONR-funded projects (Mukerji, 1989, p. 56).

Presumably NATO oceanography was also politics by other means as it helped to address differences between some of its members; the US and France especially. In 1965 Charles De Gaulle famously withdrew the French fleet from NATO command (Bozo, 1998). Although the episode might have represented an obstacle to defence integration, oceanography was accorded a special status and French oceanographers carried on working within the SACLANTCEN and the ORC. The US and French governments effectively sanctioned the existence of a domain of common interests in an otherwise fraught relation in which ‘they couldn't agree on anything else’ (cit. in Mukerji, 1989, p. 96). It notably resulted, in 1973, in the French American Mid-Ocean Undersea Study (FAMOUS. See Oreskes, 2003).

One final issue that this paper helps to re-consider, especially in light of the present attention to the origins of environmental analysis, is the legacy to Cold War science. Ronald Doel has argued that since the 1960s two distinct ‘environmental sciences’ existed: one was military-driven and the other biology-centred; one motivated by military-operational needs and the other ecology focussed; one accustomed to military sponsorship and the other critical of its implications (Doel, 2003, p. 653). This paper shows that such a division was decisive in shaping NATO oceanography as demonstrated by the funding imbalance between physical oceanography and marine biology. Yet, it also suggests that physical oceanography left a long-lasting imprint on modern environmental studies. The sub-committee did not survive long enough to carry out air-sea interaction research, but revealed the exchanges between atmosphere and oceans to be one of the earth's key environmental features thus paving the way for modern environmental analyses that emphasized this systemic co-ordination (see for instance Lovelock, 1979). NATO's transition to environmentalism accelerated the sub-committee's fall, but the research groups that replaced it inherited an emphasis on monitoring as a key feature of environmental analysis. Tools such as Anderaa's current meter found widespread application in the study of marine eco-systems, whereas the idea of sea-based monitoring array was applied more broadly to the study of water pollution. Thus if during the Cold War the buoy functioned primarily as an enemy tracking tool; it eventually became a key feature of what we now call—in an interesting merger of old terms and new priorities- ‘environmental surveillance.’

## NOTES

2 US Navy Rear-Admiral J. R. Jaap, ‘Collaboration Amongst NATO nations,’ Statement made at the Meeting of the NATO Naval Steering Group, 2–3 May 1961, Confidential, AC/141- R/6, NATO.

3 Ibid.

4 The study is based on archival collections from the NATO Archive, Brussels (NATO); the UK National Archive (TNA), London; George Deacon's papers at the National Oceanography Library, Southampton, UK (NOL); Geophysical Institute's papers at the University of Bergen, Bergen, Norway (BER). When NATO documents are copies retrieved from other archives, the provenance appears in brackets.

5 AFCP agreement, 17 May 1957 in ‘Anglo-French Collaboration in Military Oceanography,’ DEFE 69/104, TNA.

6 Directorate of Scientific Intelligence, *Oceanography and Defence in the USSR, 1956–1958* (London: Ministry of Defence, 1959), copy in DEFE 44/27, TNA.

7 The three wise men were the foreign ministers of Italy (Gaetano Martino); Norway (Halvard Lange) and Canada (Lester Pearson).

8 T. Parker, ‘Invited Statement on Matters of Defence Science to the 1st SC meeting of 26–28 March,’ Secret, 6 May 1958, AC/137-D/10, NATO.

9 K. M. Gentry, ‘Statement of future trends in anti-submarine warfare,’ Cosmic Top Secret, 17 July 1958 in AC/137-D20, NATO.

10 Ibid.

11 Ibid.

12 Norman Ramsey, ‘The Working Group on preparations for a NATO Deep-Sea Oceanographic Expedition,’ 1958, Deacon Papers, Box 87, NOL.

13 Ramsey's note in ‘The Working Group on preparations for a NATO Deep-Sea Oceanographic Expedition,’ 1958, Deacon Papers, Box 87, NOL.

14 N. Ramsey, ‘Recommendations of Ad Hoc Meeting of Experts on Oceanography,’ 3 March 1959, AC/137-D/37, NATO. Iselin did not attend this meeting and was replaced by WHOI's Bostwick H. Ketchum.

15 C. Iselin to G. Deacon, 10 April 1959 and G. Deacon, Speech at the ASW meeting, 27 April 1959 in Deacon papers, Box 87, NOL.

16 H. A. Sargeant, NATO Deputy Science Adviser to G. Deacon, 27 April 1959, Deacon Papers, Box 87, NOL.

17 H. West-Burnham, Office of Science Adviser, Proposals on Particular Defence Problems, 27 July 1959, Restricted, AC/137-D/46, NATO.

18 Statement by the US representative at the SC meeting of 10 September 1960, AC/13-R/8, NATO.

19 Although the logistic support through vessels drew heavily on resources, this was quantified only in days. NATO ORC, ‘Activities of the NATO Subcommittee of Oceanographic Research, 1959–1964,’ Bergen, January 1965, BER.

20 The panel was formed by six scientists appointed by the NATO Division of Scientific Affairs.

21 Bruun died in 1961 following his appointment as chairman of the Intergovernmental Oceanographic Commission. Minutes of ORC Meeting, 3 November 1959, AC/137-D/50, NATO; NATO, ‘Activities of the NATO Subcommittee of Oceanographic Research, 1959–1964,’ Bergen, January 1965, BER, 2.

22 Their total cost between 1960 and 1963 was in the range of $250k. Meeting of the Advisory Panel on the Research Grants Programme. 20. April 1964, AC/137-D/216 and Report of the 2nd ORC meeting, 23–24 May 1960, AC/137-D/66, NATO.

23 ORC Progress Reports, 27 September 1963, AC/137-D/187, NATO; ORC Extended Progress Report, 18 February 1964, AC/137-D/208. H. Mosby, ‘Proposal on Current Measurements in the Faroe-Shetland Channel and in the Straits of Gibraltar,’ 31 December 1959, BER, 5.

24 U. Stefánsonn to G. Deacon, 6 October 1959, Deacon Papers, Boxe 87, NOL.

25 Essentials of the Project Supported by the NATO Research Grant. Turkey, 12 July 1962, Deacon Papers, Box 87, NOL.

26 Meeting of the Advisory Panel on the Research Grants Programme. 20 April 1964, AC/137-D/216, NATO.

27 Meeting of the NATO Naval Steering Group, 2–3 May 1961, Confidential, AC/141- R/6, NATO.

28 CIA, ‘Prospects for Soviet Success in Improving Detection of Submarines in Open Ocean,’ 11.1.1974 (available at: http://www.foia.cia.gov/docs/DOC_0000681962/DOC_0000681962.pdf). The existence of a Soviet buoy array was also revealed in the British press (Defence Correspondent, ‘Submarine Counters,’*The Guardian* 2 February 1965).

29 Meeting of the NATO Naval Steering Group, 2–3 May 1961, Confidential, AC/141- R/6, NATO.

30 ORC Report of Meeting, 9–11 November 1964, AC/137-D/234, NATO. D’Ancona died in 1964 and was replaced by the Dane E. Steeman Nielsen as project coordinator.

31 Meeting of the NATO Naval Steering Group, 2–3 May 1961, Confidential, AC/141- R/6, NATO.

32 Ibid.

33 Research Oceanography, unsigned UK paper presented at AFCP meeting, 21 February 1964, DEFE 69/104, TNA.

34 Policy and General Guidance for the Provision of Military Oceanographic Information Services in NATO, Appendix A, Confidential, 11 December 1970, Military Committee Report, MC 140, NATO.

35 ‘Statement of Commander Morgan on Military Oceanography,’ Appendix II to ORC meeting of 22–23 September 1967,’ AC/137-D/318, NATO (Deacon Box 87, NOL).

36 Recommendations of the Defense Research Directors Reached in Restricted Session, Secret, 29 March 1963, AC/137(DR)D/3, NATO.

37 Report by the Exploratory Group set up to investigate the Provision of Scientific and Technical Advice to the NATO Military Authorities, Confidential, AC/137(DR)D/4, 17 October 1963, NATO.

38 Provision of Scientific Advice to the NATO Military Authorities, NATO Restricted, 16 September 1963, AC/137-D/185, NATO.

39 SC minutes of meeting, 28–29 June 1963, Restricted, AC/137-R/16, NATO.

40 SC minutes of meeting, 9 December 1963, Confidential, AC/137-R/17, NATO.

41 The confrontation typified the meetings of a UK Cabinet Group on Oceanography. Meeting of the Working Group on Oceanography, 22 May 1964, CAB 124/2170, TNA.

42 SC minutes of meeting, 2 February 1967, Restricted, AC/137-R/27 (Deacon papers, Box 88, NOL).

43 ORC report, 7–8 November 1966, AC/137-D/299 and SC minutes of meeting, 29–30 May 1967, AC 137-R/28 (Deacon papers, Box 88, NOL).

44 ORC committee report of meeting, 21–22 October 1970, AC/137(OCR)-WP/4, and SC minutes of meeting, 7 June 1971, AC/137—R/40 (Deacon papers, Box 88, NOL). COBLAMED being the Combined Operation Bouée Laboratoire Mediterranean. N. J. Campbell and E. D. Goldberg, ‘History and Achievements of the Sub-Committee on Oceanographic Research and the Special Programme on Marine Science,’ NATO Annex to ASG.SEA(77)287, 1–14 (Deacon Papers, Box 89, NOL)

45 Copy of SC minutes and handwritten notes, 11 February 1965, Confidential, in ‘NATO Science Committee,’ FO 371/189409, TNA.

46 Campbell and Goldberg, ‘History and Achievements,’ 14.

47 O. Dahl and H. Mosby, Guidelines for a steering group to be responsible for the preparation of a Final Report to the Science Committee, Report 111, undated, BER.

48 H. Mosby, Tentative Plans and Evaluation for a NATO North Atlantic Platform for Air-Sea Interaction Studies, Report 106, 21 July 1969, BER.

49 Gunnar Randers, NATO Assistant Secretary for Scientific Affairs to OCR members, 30 January 1970 in Correspondence and papers , 1970–1971, H8/8, Box 88, George Deacon Papers, NOL.

50 G. Deacon to G. Randers, 2 March 1970 in Deacon Papers, Box 88, NOL. It is worth considering that this is the time when NATO also failed to establish a NATO institute of science and technology. See Krige, 2006, pp. 218–220.

51 A. Shaler, Long-Range Programme in Ocean-Atmosphere Interaction, Document NATO Unclassified 6.5.21(70)080/AJS, 31 August 1970 (Deacon Papers, Box 88, NOL).

52 ORC Minutes of Meeting, 21 October 1970, AC/137-D/425 (Deacon Papers, Box 88, NOL).

53 ORC Minutes of Meeting, 5 April 1971, AC/137-D/441 (Deacon Papers, Box 88, NOL).

54 OCR Meeting of 13 November 1972, in H8/10 OCR Agendas and Minutes of Meetings.

55 Panel on Marine Science Minutes of Meeting, 22 March 1974, AC/137-D/541 in Deacon papers, Box 89, NOL.
